# Serum 25(OH)D concentrations and atopic diseases at age 10: results from the GINIplus and LISAplus birth cohort studies

**DOI:** 10.1186/s12887-014-0286-3

**Published:** 2014-11-25

**Authors:** Nina Wawro, Joachim Heinrich, Elisabeth Thiering, Jürgen Kratzsch, Beate Schaaf, Barbara Hoffmann, Irina Lehmann, Carl-Peter Bauer, Sibylle Koletzko, Andrea von Berg, Dietrich Berdel, Jakob Linseisen

**Affiliations:** Helmholtz Centre Munich, Institute of Epidemiology 2, Ingolstaedter Landstr 1, 85764 Neuherberg, Germany; Helmholtz Centre Munich, Institute of Epidemiology 1, Neuherberg, Germany; Ludwig-Maximilians-University Munich, Dr. von Hauner Children’s Hospital, Munich, Germany; Institute of Laboratory Medicine, Clinical Chemistry, and Molecular Diagnostics, University Hospital Leipzig, Leipzig, Germany; Medical Praxis for Children, Bad Honnef, Germany; IUF Leibniz Research Institute for Environmental Medicine, Düsseldorf, Germany; UFZ Helmholtz Centre for Environmental Research, Leipzig, Germany; Department of Pediatrics, Technical University of Munich, Munich, Germany; Department of Pediatrics, Ludwig-Maximilians-University, Munich, Germany; Department of Pediatrics, Marien-Hospital, Wesel, Germany

**Keywords:** Asthma, Atopic diseases, Eczema, Allergic rhinitis, Birth cohort, Hay fever, Sensitization, Vitamin D

## Abstract

**Background:**

Vitamin D is well recognized for its role in skeletal health and its involvement in the modulation of the immune system. In the literature, controversial results are reported for atopic diseases. Thus, we investigated the association between vitamin D status and the prevalence of atopic diseases.

**Methods:**

Serum 25-hydroxy-vitamin D (25(OH)D) concentrations were measured in a sample of 2815 10-years old children from two German birth cohort studies. Self-reported physician-diagnosed eczema, hay fever or allergic rhinitis, and asthma were used as outcome variables as well as specific IgE positivity against common allergens. We applied logistic regression models, deriving adjusted odds ratio estimates (aOR) and 95% confidence intervals (CI).

**Results:**

For asthma and hay fever or allergic rhinitis, no associations existed with serum 25(OH)D concentrations. We observed a significant positive relationship between serum 25(OH)D levels and eczema at age 10 (aOR = 1.09, CI = 1.01-1.17, per 10 nmol/l increase in serum 25(OH)D levels) and for the lifetime prevalence of eczema (aOR = 1.05, CI = 1.01-1.09). Specific IgE positivity for food allergens (aOR = 1.07, CI = 1.02-1.11) and aeroallergens (aOR = 1.05, CI = 1.01-1.08) at age 10, as well as lifetime prevalence, was significantly related to the vitamin D status.

**Conclusion:**

In this study we found no indication that higher blood 25(OH)D levels are associated with decreased risk for any of the atopic outcomes in children. However, we observed a positive association of serum 25(OH)D concentrations with eczema and detectable specific IgE. Due to the given limitations of our study, the clinical relevance of these findings needs further clarification.

**Electronic supplementary material:**

The online version of this article (doi:10.1186/s12887-014-0286-3) contains supplementary material, which is available to authorized users.

## Background

The increasing prevalence of atopic diseases during past decades in many countries led to a large number of studies, establishing life style and environmental factors as risk factors, as well as a genetic predisposition [1]. The biologically most active vitamin D metabolite 1,25(OH)2 vitamin D (calcitriol) is known to affect immune and airway functions, which is the basis of the hypothesis that vitamin D status may be directly linked to asthma and allergic diseases [2,3]. Meanwhile, several reviews have been published summarizing the existing evidence on vitamin D and atopic diseases or asthma [4-9]. The reviews highlight the lack of consistent evidence for a causal protective association between vitamin D (i.e. plasma levels as well as supplementary dietary intake) and atopic diseases.

A unique characteristic of vitamin D is that it is mostly produced by the human body after the skin is exposed to sunlight (UVB). Actually, the contribution of vitamin D intake from habitual diet to the overall vitamin D supply is limited, while endogenous synthesis is estimated to contribute up to 90% of the bodies’ vitamin D (e.g. [10]). The serum concentration of 25-hydroxy-vitamin D (25(OH)D) is an established biomarker for determining vitamin D status (see e.g. [11]).

Litonjua & Weiss hypothesized that more time is spent indoors and consequently the exposure to sunlight is reduced, leading to vitamin D insufficiency and, given a causal relationship, to more cases of asthma and allergy. Prenatal deficiency of vitamin D may already affect the development of the fetal lung and immune system which could be worsened by a postnatal deficiency of vitamin D [12]. This hypothesis of an association between vitamin D deficiency and higher rates of atopic diseases and allergic sensitization is supported by numerous studies [13-16]. In contrast, Wjst & Hyppönen see a positive association of vitamin D with allergic rhinitis in adults, which may be explained by subtle differences in the vitamin D metabolism or sensitivity in allergic patients [17]. This controversial speculation was recently supported by increased risk for food allergy within the first two years in children with high vitamin D levels in cord blood [18] and a significant inverse association of low serum vitamin D level with eczema prevalence [19]. In this situation, our study aims to contribute to this field of strong scientific interest by analyzing the relationship of serum vitamin D status at the age of ten and the prevalence of atopic diseases at the same age or life-time prevalence of these diseases.

## Methods

### Study population

The analysis was based on samples from two German birth cohort studies, namely the German Infant Study on the Influence of Nutrition Intervention plus Air pollution and Genetics on Allergy Development (GINIplus) study and the Influence of Life-style factors on Development of the Immune System and Allergies in East and West Germany plus Air Pollution and Genetics on Allergy Development (LISAplus) study, that were recruited from 1995 to 1998 in Munich and Wesel, and from 1997 to 1999 in Munich, Wesel, Bad Honnef and Leipzig, respectively.

Briefly, participants were recruited in maternity wards, and parents were invited to fill in self-administered questionnaires. Since then, families were regularly contacted, and information on disease outcomes in the children was collected by questionnaires and medical examinations. Within the GINIplus framework, children were allocated to either an interventional study arm or an observational study arm, based on parental history of allergic diseases and consent for intervention. GINI aimed to investigate the allergy-preventive effect of 3 differently hydrolyzed infant formulas compared with a conventional cow’s milk formula in infants at risk for atopy in a randomized and double-blind design. During the strict intervention period of four month, mothers were encouraged to exclusively breast-feed and asked to do this preferably for 6 months. It was left to the mother to introduce the study formula, which was provided until the participating child was 6 months old. Mothers were asked to feed no solid foods during the first four months and weekly diaries were filled out documenting the kind of milk the child was fed. In the LISAplus study children were not preselected on parental history of allergic diseases. The LISA study aimed to investigate immunoglobulin E (IgE)-dependent allergic symptoms or diseases in the first 2 years of life as well as to determine parameters of the immune system’s maturation and activation. Both studies were approved by the local ethical committees (Bavarian Board of Physicians, University of Leipzig, Board of Physicians of North-Rhine-Westphalia), and families gave their informed consent prior to their inclusion in the study.

In the first wave about 6000 newborns were recruited within the GINI study and about 3100 newborns within the LISA study. We analyzed data of 2815 children who participated in the 10 year follow-up and for which at least data on serum 25(OH)D concentration was available. The following data from the 10-year follow-up questionnaire and medical examination was used: measured weight and height, single-parent status, and net equivalent income. The 10-year follow-up medical examinations took place from April 2005 to December 2009. Detailed descriptions of both studies were published elsewhere [20,21].

When examining the distributions of the covariates from those participating in the questionnaire at age 1 year and in the assessment at age 10 years (questionnaire and the corresponding medical examination, serum vitamin D), we found only slight changes (see Additional file 1). Solely for the distribution of ‘study’ we found a higher loss of follow-up in the observational arm of GINIplus.

### Outcome assessment

We investigated different atopic diseases, either diagnosed at the age of 10 years, or ever diagnosed since birth. All these outcomes are assessed on the basis of self-administered questionnaires data. For asthma, eczema and hay fever or rhinitis, participants were categorized as ‘cases’ at the age of 10 years if a physician first diagnosed or confirmed the diagnosis of the disease during the last 12 month before the 10 year questionnaire was filled in, thereby not taking into account any information on allergic sensitization. For the definition of ‘ever’ diagnosis of these diseases information from all questionnaires from birth up to the 10 year follow-up was used (self-reports of physician diagnosed diseases). If at least at one time point a disease was reported the participating child was treated as a case for that outcome.

For definition of allergic sensitization, the serum concentrations of specific IgE antibodies against common inhalant (SX1) or food allergens (FX5) at the age of 6 and 10 years were measured. Children with specific IgE values higher than 0.35 kU/l were regarded as sensitized (Pharmacia CAP System (Pharmacia Diagnostics, Freiburg, Germany). For the definition of ‘ever’ sensitization, at least one of the two tests for specific IgE, performed at age 6 and 10, must have been positive.

For all outcomes regarding the ‘ever diagnosis’, to minimize the number of missing observations, the status ‘missing’ was only assigned when all relevant answers to determine the status were missing. In return, this means that the status ‘non-diseased control’ was assigned even when one or more answers were missing, as long as no diagnosis by a physician was affirmed.

### Determination of serum 25(OH)D concentration

Blood samples were collected at age of 10 years during the clinical examination, centrifuged after collection, and stored frozen at –80° until assayed for vitamin D. Only one measurement per sample was conducted.

Total serum 25(OH)D concentration was determined by Roche Vitamin D total on the fully automated Modular system (E170, Roche Diagnostics, Mannheim, Germany). The specificity is reported by the manufacturer as 25(OH)D_2_ = 81%; 25(OH)D_3_ = 98%; 1,25(OH)_2_D_2_ = 6%; 1,25(OH)_2_D_3_ = 5%; 24,25(OH)_2_ = 121%, and the lower limit of detection as 3 ng/mL. The intra-assay CV is 2.2-6.8% for sera with levels between 8.35 - 69.6 ng/mL, the inter-assay CV as provided by the manufacturer is 3.4-13.1% for levels between 8.35-69.6 ng/mL.

### Statistical analysis

All analyses were conducted using SAS for Windows, version 9.2 (SAS Institute, Cary, N.C., USA).

It is well known, that the vitamin D status which is assessed best by serum 25(OH)D concentration, strongly depends on sunlight (UVB) exposure which induces the endogenous production of vitamin D in the skin. Therefore, we adjusted the serum 25(OH)D values for date of blood collection by fitting a non-parametric LOESS regression. Our LOESS regression explained the observed serum 25(OH)D value by the date of blood collection. Season-adjusted serum 25(OH)D values were computed by adding the overall mean to the residuals derived from the LOESS regression. The rationale behind this is that the residuals represent the remaining variation of the serum values, whereas the seasonal effect was accounted for by the date of blood collection. Adding the residuals to the overall mean of the serum values yields a well interpretable adjusted variable. These season-adjusted serum 25(OH)D values were introduced as continuous variable in the logistic regression models, or classified in quartiles based on the distribution of the entire cohort, where the first quartile served as reference category.

To assess the association between serum 25(OH)D levels and the atopic disease status we fitted logistic regression models. Minimally adjusted odds ratio estimates were obtained by adjustment for age (continuous), sex, study (GINI observational arm/GINIinterventional arm/LISA), and study location (Munich/Wesel/Bad Honnef/Leipzig). The fully adjusted logistic regression models further included the following variables: breastfeeding (exclusively breastfed/breast and formula fed/exclusively formula fed) during the first four months, child’s BMI (continuous), parental education (categorized as low/medium/high based on the highest number of school years being lower, equal or higher than 10 years), single parent status (yes/no), and parental history of atopic diseases (none/one/both parents).

Whenever the variable sex was identified as an effect modifier, e.g. the interaction term between sex and continuous 25(OH)D serum level was significantly associated with the outcome (p < 0.05), a stratified analysis was performed.

Additionally, we fitted three models comparing those that never suffered from eczema versus those who suffered transiently and those who suffered persistently.

We ran sensitivity analysis examining higher cut-points for the specific IgEs, considering net equivalent income and by performing a complete case analysis to examine a possible influence of missing observations on the definition of the control status of the clinical outcomes.

A sub-analysis was carried out within the framework of the LISAplus study, using the monthly status on vitamin D supplementation available during the first year of life. We extended the models described above by including the main effect of length of supplementation and an interaction term thereof and 25(OH)D serum level. We investigated categories of at least 4, 6 or 12 months of supplementation, respectively.

## Results

**Figure 1:**
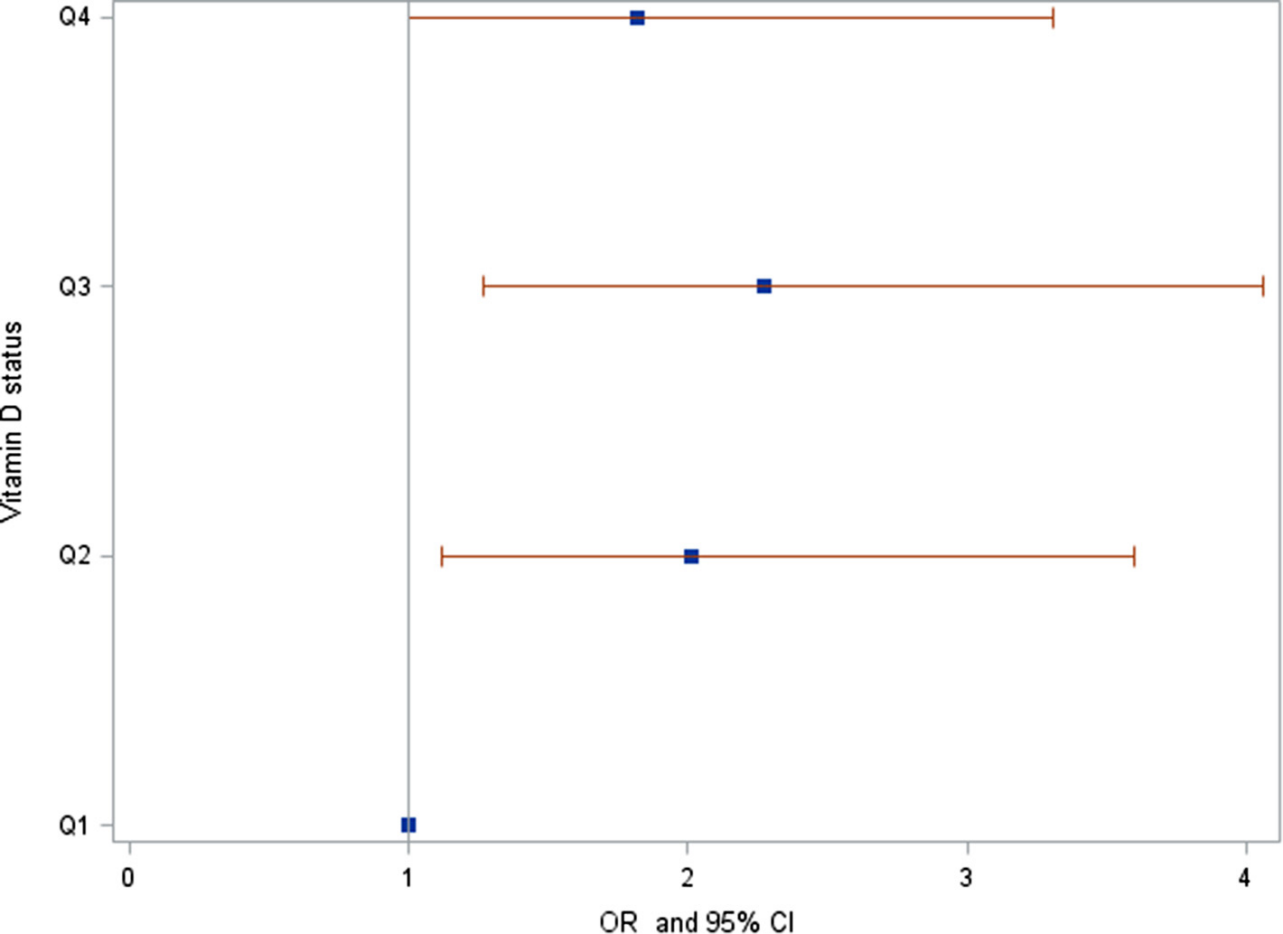
**OR estimates and 95% CI for the association of vitamin D status and eczema diagnosed at age 10 years.** OR estimates and 95% CI for the association of vitamin D status categorized in quartiles and eczema diagnosed at age 10 years. First quartile served as reference category.

Descriptive statistics on the 2815 children included in this analysis are shown in the Table 1. Tables 2, 3 and 4 show the results of the fully adjusted models fitted for all clinical and non-clinical outcomes. Results from minimally adjusted models differed only slightly (data not shown). Based on the continuous 25(OH)D variable [per 10 nmol/l] and the adjusted models, vitamin D status was significantly positively associated with the prevalence of eczema at age 10 (aOR = 1.09, CI = 1.01-1.17 per 10nmol/l) as well as with the lifetime prevalence of eczema (aOR = 1.05; CI = 1.01-1.09 per 10 nmol/l). The results of the categorized variable are given in Figure 1. Neither for hay fever, nor for allergic rhinitis an association with vitamin D status was found. For the condensed outcome ‘hay fever or rhinitis’, no association between disease prevalence and serum 25(OH)D concentration was observed, neither at the age of 10 nor for the lifetime prevalence.

**Table 1:** **Descriptive statistics of the study sample for selected covariates, total and by sex**

		**Total**		**Boys**		**Girls**	
		**n**	**(%)**	**n**	**(%)**	**n**	**(%)**
Sex	Boys	1441	(51.2)				
	Girls	1374	(48.8)				
Study	GINIplus observation	835	(29.7)	411	(28.5)	424	(30.9)
	GINIplus intervention	923	(32.8)	463	(32.1)	460	(33.5)
	LISAplus	1057	(37.6)	567	(39.4)	490	(35.7)
Study location	Munich	1553	(55.2)	799	(55.5)	754	(54.9)
	Wesel	836	(29.7)	417	(28.9)	419	(30.5)
	Bad Honnef	148	(5.3)	76	(5.3)	72	(5.2)
	Leipzig	278	(9.9)	149	(10.3)	129	(9.4)
Parental education	Low	160	(5.9)	86	(6.2)	74	(5.6)
	Medium	706	(26.0)	364	(26.3)	342	(25.7)
	High	1850	(68.1)	936	(67.5)	914	(68.7)
Single parent family	Yes	328	(11.8)	167	(11.8)	161	(11.9)
	No	2443	(88.2)	1254	(88.3)	1189	(88.1)
Parental history of atopic diseases	0	1106	(42.7)	563	(42.8)	543	(42.7)
	1	1124	(43.4)	575	(43.7)	549	(43.2)
	2	358	(13.8)	179	(13.6)	179	(14.1)
		**mean**	**(sd)**	**mean**	**(sd)**	**mean**	**(sd)**
Age		10.2	(0.4)	10.2	(0.4)	10.2	(0.4)
BMI		17.4	(2.5)	17.4	(2.5)	17.4	(2.5)
Season adjusted vitamin D [nmol/l]		74.2	(23.3)	75.2	(23.8)	73.1	(22.8)
	**1. quartile**	**2. quartile**		**3. quartile**		**4. quartile**	
Season adjusted Vitamin D [nmol/l]	<57.9	57.9- <71.5		71.5- <87.8		87.8 or higher

**Table 2:** **Odds ratio estimates and 95% confidence intervals for the association of vitamin D status and atopic diseases diagnosed at the age of 10 years**

**Outcome: at the age of 10 years**						
**Outcome**		**OR (CI)**				
		**1. Quartile**	**2. Quartile**	**3. Quartile**	**4. Quartile**	**Continuous [per 10 nmol/l]**
Asthma **male**	Cases/Non-diseased (% Cases)	21/271 (7%)	16/309 (5%)	14/282 (5%)	15/312 (5%)	66/1174 (5%)
	Adjusted model^1^	1	0.59 (0.30;1.16)	0.57 (0.28;1.17)	0.58 (0.29;1.17)	0.92 (0.82;1.03)
Asthma **female**	Cases/Non-diseased (% Cases)	4/301 (1%)	8/290 (3%)	8/285 (3%)	9/280 (3%)	29/1156 (2%)
	Adjusted model^1^	1	2.20 (0.66;7.50)	2.07 (0.61;7.05)	2.58 (0.76;8.57)	1.12 (0.97;1.30)
Eczema	Cases/Non-diseased (% Cases)	18/569 (3%)	36/567 (6%)	38/536 (7%)	31/580 (5%)	123/2252 (5%)
	Adjusted model^2^	1	2.01* (1.12;3.60)	2.27* (1.27;4.06)	1.82 (1.00;3.31)	1.09* (1.01;1.17)
Hay fever or allergic rhinitis	Cases/Non-diseased (% Cases)	57/536 (10%)	77/539 (13%)	66/513 (11%)	67/548 (11%)	267/2136 (11%)
	Adjusted model^2^	1	1.28 (0.89;1.86)	1.12 (0.76;1.64)	1.07 (0.73;1.57)	0.98 (0.93;1.04)

^1^Outcome = Vitamin D concentration + age + study + breastfeeding + BMI + highest parental education + family history.

^2^Outcome = Vitamin D concentration + age + sex + location of study + study + breastfeeding + BMI + highest parental education + single parent status + family history.

The OR estimates and 95% CI for the association of vitamin D status (serum 25(OH)D concentration in quartiles or as continuous variable [10 nmol/l]) and atopic diseases diagnosed at the age of 10 years are displayed. The asterisk indicate significant results at p < 0.05.

**Table 3:** **Odds ratio estimates and 95% confidence intervals for the association of vitamin D status and ever diagnosed atopic diseases**

**Outcome: ever diagnosis**						
**Outcome**		**OR (CI)**				
		**1. Quartile**	**2. Quartile**	**3. Quartile**	**4. Quartile**	**Continuous [per 10 nmol/l]**
Asthma **male**	Cases/Non-diseased (% Cases)	34/265 (11%)	35/297 (11%)	25/282 (8%)	39/303 (11%)	133/1147 (10%)
	Adjusted model^1^	1	0.84 (0.50;1.40)	0.65 (0.37;1.13)	0.97 (0.59;1.61)	1.00 (0.92;1.07)
Asthma **female**	Cases/Non-diseased (% Cases)	15/295 (5%)	22/281 (7%)	22/289 (7%)	17/278 (6%)	76/1143 (6%)
	Adjusted model^1^	1	1.54 (0.78;3.06)	1.48 (0.74;2.93)	1.21 (0.59;2.50)	1.02 (0.92;1.13)
Eczema	Cases/Non-diseased (% Cases)	173/430 (29%)	196/430 (31%)	184/420 (30%)	195/433 (31%)	748/1713 (30%)
	Adjusted model^2^	1	1.12 (0.87;1.44)	1.09 (0.84;1.40)	1.14 (0.89;1.46)	1.05* (1.01;1.09)
Hay fever or allergic rhinitis	Cases/Non-diseased (% Cases)	99/504 (16%)	119/507 (19%)	117/487 (19%)	121/507 (19%)	456/2005 (19%)
	Adjusted model^2^	1	1.13 (0.84;1.53)	1.16 (0.86;1.57)	1.15 (0.85;1.56)	1.02 (0.98;1.07)

^1^Outcome = Vitamin D concentration + age + study + breastfeeding + BMI + highest parental education + family history.

^2^Outcome = Vitamin D concentration + age + sex + location of study + study + breastfeeding + BMI + highest parental education + single parent status + family history.

The OR estimates and 95% CI for the association of vitamin D status (serum 25(OH)D concentration in quartiles or as continuous variable [10 nmol/l]) and ever diagnosed atopic diseases are displayed. The asterisk indicate significant results at p < 0.05.

**Table 4:** **Odds ratios and 95% confidence intervals for the association of vitamin D status and allergic sensitization at the age of 10 years and ever sensitization**

**Outcome**		**Outcome: at the age of 10 years**				
		**OR (CI)**				
		**1. Quartile**	**2. Quartile**	**3. Quartile**	**4. Quartile**	**Continuous [per 10 nmol/l]**
Spec. IgE (food allergen mixture)	Cases/Non-diseased (% Cases)	100/503 (17%)	98/528 (16%)	123/481 (20%)	130/498 (21%)	451/2010 (18%)
	Adjusted model^1^	1	0.91 (0.67;1.25)	1.25 (0.93;1.69)	1.30 (0.97;1.75)	1.07* (1.02;1.11)
Spec. IgE (inhalant allergen mixture)	Cases/Non-diseased (% Cases)	220/383 (36%)	244/382 (39%)	238/366 (39%)	268/360 (43%)	970/1491 (39%)
	Adjusted model^1^	1	1.08 (0.85;1.36)	1.09 (0.86;1.38)	1.24 (0.98;1.57)	1.05* (1.01;1.08)
**Outcome**		**Outcome: ever diagnosis**				
		**OR (CI)**				
		**1. Quartile**	**2. Quartile**	**3. Quartile**	**4. Quartile**	**Continuous [per 10 nmol/l]**
Spec. IgE (food allergen mixture)	Cases/Non-diseased (% Cases)	121/351 (26%)	118/377 (24%)	137/352 (28%)	157/346 (31%)	533/1426 (27%)
	Adjusted model^1^	1	0.92 (0.68;1.23)	1.13 (0.84;1.51)	1.33 (1.00;1.77)	1.06* (1.02;1.11)
Spec. IgE (inhalant allergen mixture)	Cases/Non-diseased (% Cases)	229/270 (46%)	250/275 (48%)	243/276 (47%)	281/239 (54%)	1003/1060 (49%)
	Adjusted model^1^	1	1.06 (0.83;1.37)	1.02 (0.79;1.32)	1.34* (1.04;1.73)	1.05* (1.01;1.10)

^1^Outcome = Vitamin D concentration + age + sex + location of study + study + breastfeeding + BMI + highest parental education + single parent status + family history.

The OR estimates and 95% CI for the association of vitamin D status (serum 25(OH)D concentration in quartiles or as continuous variable [10 nmol/l]) and allergic sensitization (specific serum IgE concentrations) at the age of 10 years and ever sensitization (specific serum IgE concentrations that were measured at the age of 6 years and 10 years). The asterisk indicate significant results at p < 0.05.

For asthma, no association with vitamin D status was observed. Due to low numbers, neither the study location nor the single-parent status could be taken into account in the gender-specific analysis. Neither in boys nor in girls vitamin D status and asthma were significantly associated.

Regarding allergic sensitization (Table 4), significant associations exist between vitamin D status and specific IgE against food allergens (aOR = 1.07; CI = 1.02-1.11 at the age of 10, aOR = 1.06; CI = 1.02-1.11 for ever sensitization). A similar result was observed for serum concentrations of specific IgE against inhalant allergens: the adjusted OR was 1.05 (CI = 1.01-1.08) at the age of 10 years and aOR = 1.05 (CI = 1.01-1.10) for ever sensitization.

Table 5 shows the results from the additional analysis of eczema. A significant association of vitamin D status at the age of 10 and prevalence of eczema was confirmed by the results of the continuous 25OHD variable only for the persistent cases compared to ‘non diseased children’ (aOR = 1.09; CI = 1.03-1.16). In both other settings (i.e., early or late transient cases), no significant associations existed.

**Table 5:** **Odds ratio estimates and 95% confidence intervals for the association of vitamin D status and eczema, stratified by age of onset and duration of eczema**

		**OR (CI)**				
		**1. Quartile**	**2. Quartile**	**3. Quartile**	**4. Quartile**	**Continuous [per 10 nmol/l]**
Never vs. only when younger than 6 years	Cases/Non-diseased (% Cases)	102/430 (19%)	107/430 (20%)	91/420 (18%)	104/433 (19%)	404/1713 (19%)
	Adjusted model^1^	1	1.06 (0.78;1.44)	0.93 (0.68;1.28)	1.04 (0.76;1.43)	1.02 (0.97;1.07)
Never vs. only when 6 years or older	Cases/Non-diseased (% Cases)	19/430 (4%)	25/430 (5%)	32/420 (7%)	20/433 (4%)	96/1713 (5%)
	Adjusted model^1^	1	1.28 (0.69;2.39)	1.70 (0.94;3.08)	1.08 (0.56;2.08)	1.04 (0.94;1.14)
Never vs. persistent cases	Cases/Non diseased (% Cases)	52/430 (11%)	64/430 (13%)	61/420 (13%)	70/433 (14%)	247/1713 (13%)
	Adjusted model^1^	1	1.16 (0.78;1.722)	1.16 (0.78;1.74)	1.36 (0.92;2.01)	1.09^*^ (1.03;1.16)

^1^Outcome = Vitamin D concentration + age + sex + location of study + study + breastfeeding + BMI + highest parental education + single parent status + family history.

The OR estimates and 95% CI for the association of vitamin D status (serum 25(OH)D concentration [10 nmol/l]) and eczema, stratified by age of onset and duration of eczema (never versus early onset but transient, late onset but transient and persistent cases) are displayed. The asterisk indicate significant results at p < 0.05.

Considering additionally the net equivalent income, no substantial changes regarding associations of the vitamin D status and the outcomes were observed, even though the sample sizes were reduced. When rising the cut-point of the specific IgE serum concentration values from >0.35 kU/l to >0.7 kU/l for models at age 10, the observed risk estimates did not change materially. However, the association of specific IgE against inhalant allergens and 25(OH)D level dropped below significance level (results not shown).

The sub-analysis considering vitamin D supplementation revealed no effect modification by vitamin D supplementation during the first year of life: neither the main effect nor the interaction term became significant (results not shown). This holds for all outcomes examined, but confounding by socio-economic factors cannot be ruled out.

## Discussion

We investigated associations of 25(OH)D serum levels at the age of 10 and prevalence of asthma, eczema, hay fever or allergic rhinitis, and allergic sensitization. Disease status at age 10 and lifetime prevalence of disease were analyzed as outcomes. We observed small but statistically significant associations of serum 25(OH)D levels and prevalence of eczema as well as the prevalence of allergic sensitization against common food and inhalant allergens.

Published findings regarding an association of vitamin D status and atopic outcomes are not consistent. A comparison of the results to our findings is complicated by differences in the assessment of vitamin D status (e.g. measurements in cord blood or assessment of maternal vitamin D status during pregnancy), in study designs, the age of participants or the definition of the outcomes.

For our finding of a slightly increased prevalence for eczema with high serum 25(OH)D concentrations, only one directly comparable result could be identified that was as well derived in a population based cohort of children and included serum 25(OH)D concentration measurement. This study also found a decreased prevalence of atopic eczema with low serum 25(OH)D concentrations in children aged 0-17 years [19]. Also, a positive association between vitamin D intake and atopic manifestations was described [22]. In a study using cord blood vitamin D levels, no relationship with atopic eczema in children was found [18], but opposing results were reported by others [15,16]. The link of the severity of atopic dermatitis and vitamin D has been confirmed repeatedly (see e.g. [23] or more recently [24]). Benson et al. suggested that the inconsistent connection between vitamin D and allergic skin diseases may be due to a bimodal or gender-specific association [25]; this is not supported by our results.

We could not confirm any association of serum 25(OH)D concentration and hay fever or allergic rhinitis. Published studies with direct assessment of serum 25(OH)D concentration reported similar null findings (e.g. in a birth cohort [26]), but as well an increased prevalence of allergic rhinitis across quartiles of 25(OH)D concentrations [17]. Furthermore a marginally significant increase of allergic rhinitis prevalence in the group with highest vitamin D cord blood was reported [27].

Studies assessing vitamin D status of the mothers or performing measurements in cord blood samples are inconclusive regarding the existence of an association between vitamin D status and risk of asthma [28-30]. Significant associations of asthma and vitamin D have been reported by other studies that directly measured serum 25(OH)D concentrations, although the direction of the associations remains inconclusive. For example, associations that change direction with increasing age, from positive to inverse, between serum 25(OH)D levels and prevalence of asthma were reported [31]. Significantly lower serum 25(OH)D levels among the asthmatic cases in a case-control study conducted in Qatar in children [32] and an inverse association of serum 25(OH)D concentrations and asthma in children [33] have been published. Low serum 25(OH)D levels at age 6 were reported as significant predictors for asthma-associated phenotypes at age 14 in a birth cohort [26]. Recently, an increased odds of asthma has been reported for low vitamin D levels in children with atopy [34]. Our results show no such association.

Regarding allergic sensitization, we reported a significant positive association of serum 25(OH)D concentrations and specific IgE against common food allergens and against inhalant allergens, respectively. Published results based on direct assessment of serum 25(OH)D concentrations were either supportive of our findings, e.g. [26,35] or are contradictory (e.g. [36]). However, results based on cord blood 25(OH)D concentrations were contradictory [18,27,37]. In Asian children aged 5-18 years, no association of serum 25(OH)D status with asthma, rhinitis, eczema, atopy or total serum IgE was found [38].

### Strength and limitations

Measurement of vitamin D status is challenging as results vary strongly with the methods applied [39]. The 25(OH)D levels analyzed in serum samples of children participating in the GINIplus and LISAplus study are considerably higher than reported elsewhere for this age group. This might be due to the assay applied with a comparably low specificity for 25(OH)D and a high cross-reactivity (see *Methods* section). We used the serum concentration for ranking (in quartiles) and as a continuous variable (p trend). However, we avoided interpretation of serum concentrations in terms of sufficiency of vitamin D supply that requires the application of cut-off values.

Our evaluation is based on serum 25(OH)D concentrations measured in serum samples obtained at one time point, about 10 years after birth. With the applied LOESS regression we controlled adequately on a continuous scale for the seasonal variation in vitamin D status; in other studies often only the date or season of blood collection was included as a confounder in the analysis. All relevant potential confounders were included in the adjusted models.

The cross-sectional analysis at age 10 is suitable to investigate the association of vitamin D status and atopic diseases. Although the occurrence of atopic diseases from birth up to age 10 years (‘ever diagnosis’) was included in our evaluation, serum 25(OH)D concentrations were analyzed in serum samples obtained at age 10 years. Thus, reverse causation effects cannot be ruled out (see e.g. [7]). There is little known about the stability of vitamin D levels in children with a fast changing lifestyle. Our data suggest that the lifetime prevalence of eczema as estimated at the age of 10 years represents predominantly the persistent cases of eczema.

Our analysis of clinical endpoints is based on self-reported physician-based diagnoses and therefore at risk of reporting bias. Furthermore eczema, allergic rhinitis, and asthma are each inexact, clinical diagnoses which might amplify this imprecision. The definition of the ‘non-diseased controls’ as described in the Methods section is debatable. Among the ‘non-diseased controls’ may be cases who missed to report a physician diagnosis. The sensitivity analysis performed included only complete cases in the definition of the control status. This led to a remarkably reduced number of controls. However, no substantial changes regarding the direction of the OR estimates were observed, supporting the validity of the approach taken. We included a non-responder analysis showing that a selective lost for follow up between the study population and the recruited newborns is limited.

## Conclusion

Our main findings are a lack of inverse association between serum 25(OH)D concentrations and clinically diagnosed atopic diseases, but a positive association with the prevalence of eczema. The associations were significant for the categorical and continuous variables of vitamin D and eczema. However, we see no dose-response effect. For allergic sensitization we noted a small but statistically significant association. Even though this association has limited clinical relevance, it gives hint on a specific role of vitamin D on pathophysiologically important pathways, especially on the regulation of innate and adaptive immune system as shown in experimental studies (e.g. [40]). Due to the study design, the results have to be interpreted carefully. Especially, we cannot rule out the possibility of reverse causality.

## Additional file

Additional file 1:
**Non-responder Analysis.** We provide a non-responder analysis, showing the distribution of relevant covariates at different time points. Further covariates included in the analysis (BMI, age, net equivalent income, single parent status) refer to the recording at age 10 not at baseline and are therefore not included in the table.

## Abbreviations

GINIplus: German infant study on the influence of nutrition intervention plus air pollution and genetics on allergy development
LISAplus: Influence of life-style factors on development of the immune system and allergies in east and west germany plus air pollution and genetics on allergy development
25(OH)D: 25-hydroxy vitamin D
UVB: Ultra violet B
kU/l: Kilo units per liter
nmol/l: Nanomols per liter
IgE: Immunoglobulin E
BMI: Body mass index
aOR: Adjusted odds ratio
CI: Confidence interval
sd: Standard deviation
Q: Quartile

## References

[1] von Mutius E (2000). The environmental predictors of allergic disease. J Allergy Clin Immunol.

[2] Muehleisen B, Gallo RL (2013). Vitamin D in allergic disease: shedding light on a complex problem. J Allergy Clin Immunol.

[3] Krstic G (2011). Asthma prevalence associated with geographical latitude and regional insolation in the United States of America and Australia. PLoS One.

[4] Hollams EM (2012). Vitamin D and atopy and asthma phenotypes in children. Curr Opin Allergy Clin Immunol.

[5] Searing DA, Leung DY (2010). Vitamin D in atopic dermatitis, asthma and allergic diseases. Immunol Allergy Clin North Am.

[6] Mutgi K, Koo J (2013). Update on the role of systemic vitamin d in atopic dermatitis. Pediatr Dermatol.

[7] Jones AP, Tulic MK, Rueter K, Prescott SL (2012). Vitamin d and allergic disease: sunlight at the end of the tunnel?. Nutrients.

[8] Litonjua AA (2012). Vitamin D deficiency as a risk factor for childhood allergic disease and asthma. Curr Opin Allergy Clin Immunol.

[9] Paul G, Brehm JM, Alcorn JF, Holguin F, Aujla SJ, Celedon JC (2012). Vitamin D and asthma. Am J Respir Crit Care Med.

[10] Bozzetto S, Carraro S, Giordano G, Boner A, Baraldi E (2012). Asthma, allergy and respiratory infections: the vitamin D hypothesis. Allergy.

[11] Zerwekh JE (2008). Blood biomarkers of vitamin D status. Am J Clin Nutr.

[12] Litonjua AA, Weiss ST (2007). Is vitamin D deficiency to blame for the asthma epidemic?. J Allergy Clin Immunol.

[13] Camargo CA, Clark S, Kaplan MS, Lieberman P, Wood RA (2007). Regional differences in EpiPen prescriptions in the United States: the potential role of vitamin D. J Allergy Clin Immunol.

[14] Osborne NJ, Ukoumunne OC, Wake M, Allen KJ (2012). Prevalence of eczema and food allergy is associated with latitude in Australia. J Allergy Clin Immunol.

[15] Jones AP, Palmer D, Zhang G, Prescott SL (2012). Cord blood 25-hydroxyvitamin D3 and allergic disease during infancy. Pediatrics.

[16] Baiz N, Dargent-Molina P, Wark JD, Souberbielle JC, Annesi-Maesano I (2014). Cord serum 25-hydroxyvitamin D and risk of early childhood transient wheezing and atopic dermatitis. J Allergy Clin Immunol.

[17] Wjst M, Hypponen E (2007). Vitamin D serum levels and allergic rhinitis. Allergy.

[18] Weisse K, Winkler S, Hirche F, Herberth G, Hinz D, Bauer M, Roder S, Rolle-Kampczyk U, von Bergen M, Olek S, Sack U, Richter T, Diez U, Borte M, Stangl GI, Lehmann I (2013). Maternal and newborn vitamin D status and its impact on food allergy development in the German LINA cohort study. Allergy.

[19] Heimbeck I, Wjst M, Apfelbacher CJ (2013). Low vitamin D serum level is inversely associated with eczema in children and adolescents in Germany. Allergy.

[20] von Berg A, Koletzko S, Grubl A, Filipiak-Pittroff B, Wichmann HE, Bauer CP, Reinhardt D, Berdel D (2003). The effect of hydrolyzed cow’s milk formula for allergy prevention in the first year of life: the German infant nutritional intervention study, a randomized double-blind trial. J Allergy Clin Immunol.

[21] Zutavern A, Rzehak P, Brockow I, Schaaf B, Bollrath C, von Berg A, Link E, Kraemer U, Borte M, Herbarth O, Wichmann HE, Heinrich J (2007). Day care in relation to respiratory-tract and gastrointestinal infections in a German birth cohort study. Acta Paediatr.

[22] Back O, Blomquist HK, Hernell O, Stenberg B (2009). Does vitamin D intake during infancy promote the development of atopic allergy?. Acta Derm Venereol.

[23] Peroni DG, Piacentini GL, Cametti E, Chinellato I, Boner AL (2011). Correlation between serum 25-hydroxyvitamin D levels and severity of atopic dermatitis in children. Br J Dermatol.

[24] Wang SS, Hon KL, Kong AP, Pong HN, Wong GW, Leung TF (2014). Vitamin D deficiency is associated with diagnosis and severity of childhood atopic dermatitis. Pediatr Allergy Immunol.

[25] Benson AA, Toh JA, Vernon N, Jariwala SP (2012). The role of vitamin D in the immunopathogenesis of allergic skin diseases. Allergy.

[26] Hollams EM, Hart PH, Holt BJ, Serralha M, Parsons F, de Klerk NH, Zhang G, Sly PD, Holt PG (2011). Vitamin D and atopy and asthma phenotypes in children: a longitudinal cohort study. Eur Respir J.

[27] Rothers J, Wright AL, Stern DA, Halonen M, Camargo CA (2011). Cord blood 25-hydroxyvitamin D levels are associated with aeroallergen sensitization in children from Tucson, Arizona. J Allergy Clin Immunol.

[28] Pike KC, Inskip HM, Robinson S, Lucas JS, Cooper C, Harvey NC, Godfrey KM, Roberts G (2012). Maternal late-pregnancy serum 25-hydroxyvitamin D in relation to childhood wheeze and atopic outcomes. Thorax.

[29] Camargo CA, Ingham T, Wickens K, Thadhani R, Silvers KM, Epton MJ, Town GI, Pattemore PK, Espinola JA, Crane J (2011). Cord-blood 25-hydroxyvitamin D levels and risk of respiratory infection, wheezing, and asthma. Pediatrics.

[30] Gale CR, Robinson SM, Harvey NC, Javaid MK, Jiang B, Martyn CN, Godfrey KM, Cooper C (2008). Maternal vitamin D status during pregnancy and child outcomes. Eur J Clin Nutr.

[31] van Oeffelen AA, Bekkers MB, Smit HA, Kerkhof M, Koppelman GH, Haveman-Nies A, AD v d, Jansen EH, Wijga AH (2011). Serum micronutrient concentrations and childhood asthma: the PIAMA birth cohort study. Pediatr Allergy Immunol.

[32] Bener A, Ehlayel MS, Tulic MK, Hamid Q (2012). Vitamin D deficiency as a strong predictor of asthma in children. Int Arch Allergy Immunol.

[33] Alyasin S, Momen T, Kashef S, Alipour A, Amin R (2011). The relationship between serum 25 hydroxy vitamin d levels and asthma in children. Allergy Asthma Immunol Res.

[34] Checkley W, Robinson CL, Baumann LM, Hansel NN, Romero K, Pollard SL, Wise RA, Gilman RH, Mougey E, Lima JJ, PURA study investigators: **25-hydroxy vitamin D levels are associated with childhood asthma in a population-based study in Peru.** *Clin Exp Allergy* 2014, doi:10.1111/cea.12311. [Epub ahead of print]. 10.1111/cea.12311 PMC4176605 24666565

[35] Hypponen E, Berry DJ, Wjst M, Power C (2009). Serum 25-hydroxyvitamin D and IgE - a significant but nonlinear relationship. Allergy.

[36] Allen KJ, Koplin JJ, Ponsonby AL, Gurrin LC, Wake M, Vuillermin P, Martin P, Matheson M, Lowe A, Robinson M, Tey D, Osborne NJ, Dang T, Tina Tan HT, Thiele L, Anderson D, Czech H, Sanjeevan J, Zurzolo G, Dwyer T, Tang ML, Hill D, Dharmage SC (2013). Vitamin D insufficiency is associated with challenge-proven food allergy in infants. J Allergy Clin Immunol.

[37] Liu X, Wang G, Hong X, Wang D, Tsai HJ, Zhang S, Arguelles L, Kumar R, Wang H, Liu R, Zhou Y, Pearson C, Ortiz K, Schleimer R, Holt PG, Pongracic J, Price HE, Langman C, Wang X (2011). Gene-vitamin D interactions on food sensitization: a prospective birth cohort study. Allergy.

[38] Yao TC, Tu YL, Chang SW, Tsai HJ, Gu PW, Ning HC, Hua MC, Liao SL, Tsai MH, Chiu CY, Lai SH, Yeh KW, Huang JL (2014). Suboptimal vitamin D status in a population-based study of Asian children: prevalence and relation to allergic diseases and atopy. PLoS One.

[39] Linseisen J, Abbas S, Akesson B, Lodz MP (2007). Measurement of Serum 25-Hydroxycholecalciferol as Marker of Vitamin D Status. Dietary Vitamins, Polyphenols, Selenium And Probiotics: Biomarkers of Exposure and Mechanisms of Anticarcinogenic Action.

[40] Prietl B, Treiber G, Pieber TR, Amrein K (2013). Vitamin D and immune function. Nutrients.

