# Supplementary material for: Serum 25(OH)D concentrations and atopic diseases at age 10: results from the GINIplus and LISAplus birth cohort studies
Source: BMC Pediatr. 2014 Nov 25;14:286. doi: 10.1186/s12887-014-0286-3 (PMC4251945; doi:10.1186/s12887-014-0286-3)
Supplement: Additional file 1: — Non-responder Analysis. We provide a non-responder analysis, showing the distribution of relevant covariates at different time points. Further covariates included in the analysis (BMI, age, net equivalent income, single parent status) refer to the recording at age 10 not at baseline and are therefore not included in the table. [file 12887_2014_286_MOESM1_ESM.pdf]

Covariates		Baseline questionnaire N=9086 (+1*)		Questionnaire at age 1 year N=7406		Questionnaire at age 10 years N=5078		Questionnaire and medical examination at age 10 years N=3121		Questionnaire, medical examination and serum vitamin D available N=2815	
		**N**	**%**	**N**	**%**	**N**	**%**	**N**	**%**	**N**	**%**
Sex	Male	4576	51.3	3826	51.7	2590	51.0	1588	50.9	1441	51.2
	Female	4349	48.7	3580	48.3	2488	49.0	1533	49.1	1374	48.8
	missing	*161*									
Location of study	Munich	4414	48.6	3585	48.4	2670	52.6	1685	54.0	1553	55.2
	Leipzig	976	10.7	820	11.1	435	8.6	338	10.8	278	9.9
	Bad Honnef	306	3.4	276	3.7	207	4.1	159	5.1	148	5.3
	Wesel	3390	37.3	2725	36.8	1766	34.8	939	30.1	836	29.7
Study	GINI Observ	3739	41.2	2812	38.0	1866	36.7	907	29.1	835	29.7
	GINI Interv	2252	24.8	1859	25.1	1451	28.6	1014	32.5	923	32.8
	LISA	3095 (+1)	34.1	2735	36.9	1761	34.7	1200	38.4	1057	37.6
# parents w atopic diseases	0			3399	49.2	2202	47.2	1239	43.2	1106	42.7
	1			2669	38.6	1870	40.1	1236	43.1	1124	43.4
	2			845	12.2	591	12.7	395	13.8	358	13.8
	missing			*493*		*415*		*251*		*227*	
Highest parental education	<10 y			567	8.3	348	6.5	170	5.6	160	5.9
	10 y			1940	28.5	1314	26.4	802	26.6	706	26.0
	>10y			4309	63.2	3236	67.0	2041	67.7	1850	68.1
	missing			*590*		*180*		*108*		*99*	
Breast feeding during first 4 month	Breast fed (Bf)			3910	53.6	2791	56.9	1782	59.2	1606	59.3
	Bf+ Ff			2583	35.4	1639	33.4	990	32.9	893	33.0
	Formular fed (Ff)			805	11.0	472	9.6	236	7.8	209	7.7
	missing			*108*		*176*		*113*		*107*	

*1 Total drop out: 3096 children were recruited within LISA, consent was withdrawn from 1 child
